# One cell, multiple roles: contribution of mesenchymal stem cells to tumor development in tumor microenvironment

**DOI:** 10.1186/2045-3701-3-5

**Published:** 2013-01-21

**Authors:** Xue Yang, Jing Hou, Zhipeng Han, Ying Wang, Chong Hao, Lixin Wei, Yufang Shi

**Affiliations:** 1 Tumor Immunology and Gene Therapy Center, Eastern Hepatobiliary Surgery Hospital, the Second Military Medicial University, 225 Changhai Road, Shanghai 200438, China; 2 Key Laboratory of Stem Cell Biology, Institute of Health Sciences, Shanghai Jiao Tong University School of Medicine (SJTUSM) & Shanghai Institutes for Biological Sciences (SIBS), Chinese Academy of Sciences (CAS), Shanghai 200025, China

**Keywords:** Mesenchymal stem cells (MSCs), Tumor microenvironment, Tumor growth, Metastasis

## Abstract

The discovery of tissue reparative and immunosuppressive abilities of mesenchymal stem cells (MSCs) has drawn more attention to tumor microenvironment and its role in providing the soil for the tumor cell growth. MSCs are recruited to tumor which is referred as the never healing wound and altered by the inflammation environment, thereby helping to construct the tumor microenvironment. The environment orchestrated by MSCs and other factors can be associated with angiogenesis, immunosuppression, inhibition of apoptosis, epithelial-mesenchymal transition (EMT), survival of cancer stem cells, which all contribute to tumor growth and progression. In this review, we will discuss how MSCs are recruited to the tumor microenvironment and what effects they have on tumor progression.

## Introduction

Mesenchymal stem cells (MSCs, also called as mesenchymal stromal cells) is a subset of non-hematopoietic adult stem cells which originate from mesoderm. They possess self-renew ability and multilineage differentiation into not only mesoderm-lineage, such as chondrocytes, osteocytes and adipocytes, but also ectodermic cells and endodermic cells [1-5]. MSCs exist in almost all tissues. They can be easily isolated from bone marrow, adipose, umbilical cord, fetal liver, muscle, lung and etc, and can be successfully expanded *in vitro*[6-10]. Due to lack of specific markers to define MSCs, their identification was depended on the plastic adhesion property, a panel of surface markers, including CD31, CD34, CD45, CD29, CD90, and CD105, as well as multiple differentiation potential. Together with immunosuppressive properties endowed by the inflammation in the damaged tissues, MSCs can display their tissue reparative function. Above is observed in MSC-based therapy in vigorous inflammatory diseases, however, with chronic and insufficient inflammation, MSCs cannot rescue the tissue damage, even worsening the disease. Inflammation is always associated with tumor development where tissue suffers from chronic injury. Based on the property of MSCs being recruited to injured tissues, MSCs are used to deliver anti-tumor reagent directly to tumors for cell based therapy [11-13]. However, the role of MSCs in constructing tumor microenvironment and its potential mechanisms are still controversial. Here, we will focus on the effects of MSCs on the tumorigenesis and tumor metastasis.

The “seed and soil” hypothesis was proposed by Paget in the late nineteenth century, so we can imagine how important the tumor microenvironment is. Tumor microenvironment is very complicated, and includes various cell types, lots of soluble factors, extensive neovasculature [14] and excessive extracellular matrix (ECM) deposition. The network orchestrated by tumor cells, stroma cells [15] and the soluble factors contribute to tumorigenesis, progression, metastases and reoccurrence [16].

### MSCs and other components in tumor microenvironment

Tumor microenvironment always provides essential conditions to maintain cancer stem cells/cancer initiating cells, as well as to boost the cancer cell metastasis. Distinct types of cells, including fibroblast stromal cells (also known as tumor-associated fibroblasts, TAFs) [15,17], immune cells, endothelial cells, adipocytes, and mesenchymal stem cells (MSCs) [17] contribute to tumor progression through crosstalking with each other in either direct or indirect manners. Once tumor develops, TAFs are activated to repair the never healing wound [18]. TAFs play roles in tumor stroma organization by producing plentiful ECM, meanwhile, they contribute to angiogenesis together with endothelial cells and macrophages by producing growth factors, cytokines, chemokines, matrix-degrading enzymes [19]. A growing number of researches demonstrated that blood vessels formed by endothelial cells are responsible for supplying nutrients and transporting metabolic and biological waste [20], thus tumor angiogenesis is important in many types of tumors [21]. In addition, adipocytes, another component of energy supplier, are reported to promote homing, migration and invasion of tumor cells by secreting adipokines including interleukin-8 (IL-8) and also make tumors grow rapidly by providing fatty acids [22]. Meanwhile, the immune surveillance built up by tumor-associated macrophage (TAM), NK cells, T cells, B cells, polymorphonuclear leukocytes (PMN), and dendritic cells (DCs) [1] in the tumor sites should not be ignored. They can shift the tumor immune microenvironment, thereby favoring the tumor progression, invasion, malignancy and relapse [17]. With the advent of tissue repair and immune regulatory function of MSCs, MSCs has attracted more attention to their roles in regulating tumor environment [1,23]. MSCs are recruited from remote sites into the tumor sites, therein influencing tumor microenvironment by interacting with other cell types or secreting soluble factors. In addition, MSCs are able to differentiate into several stromal cells, such as adipocytes and TAFs [1,7], which was reported in an induced gastric cancer model [24]. Striking evidence also indicated that MSCs played a critical role in tumor vasculogenesis by differentiating into pericytes and endothelial-like cells [1]. Together with cytokines [Table 1], chemokines [Table 2] and condition where they resided in, MSCs played the indispensable role in regulating different stages of tumor progression.

**Table 1:** Cytokines in tumor microenvironment and effects on tumor progression

**Cytokines**	**Cellular sources**	**Tumor growth**	**Metastasis**	**Immune response**	**References**
IL-1	macrophages, DCs, B cells, NK cells, keratinocytes, tumor cells	+	+		[25,26]
IL-2	Th1 lymphocytes	-		+	[26,27]
IL-4	Th2 lymphocytes	-		+	[28]
IL-6	T (mainly Th2) and B cells, keratinocytes and macrophages. tumor cell, fibroblast, endothelial cells	+(low concentration)		+	[25,26,29-31]
		-(high concentration)			
IL-8	tumor cells	+			[26]
IL-10	Th cells, B cells, activated monocytes, macrophages, thymocytes, keratinocytes, and tumor cells.	+	+	-	[29,32,33]
IL-11					
IL-12	APC: monocytes, macrophages, and DCs. tumor cells, neutrophils	-	-	+	[26,34-38]
IL-15	macrophages, DCs	-		+	[39]
IL-18			-		[26]
IFN-α		-		+	[40]
IFN-β		-		+	[40]
IFN-γ	T (mainly Th1)and B cells, NK cells, NKT cells, CTL macrophages, mast cells, DCs	-		+	[25,26]
TNF-α	activated macrophages, T and B cells, NK cells, tumor cells, neutrophils, fibroblasts, keratinocytes	+(low concentration)			[25,29]
		-(high concentration)			
TGF-β	T and B cells, macrophages, platelets, bone-marrow stroma, tumor cells	-		+	[25,41]
M-CSF	macrophages, endothelial cells, fibroblasts, bone-marrow stroma	+	+		[25]
GM-CSF	respiratory epithelial cells, T cells, NK cells, NKT cells, macrophages, eosinophils, endothelial cells, fibroblasts	-			[25]
MIF	macrophages, T cells, eosinophils, fibroblasts, keratinocytes, pituitary	+			[25]

Abbreviations: +, promote ; —, inhibit.

**Table 2:** Chemokines in tumor microenvironment and effects on tumor progression

**Chemokines**	**Chemokine receptors**	**Cellular sources**	**Tumor growth**	**Metastasis**	**Immune response**	**References**
CCL2	CCR2	tumor cells, macrophages, endothelial cells, TAFs	+(low concentration)	+	-	[42-46]
			-(high concentration)			
CCL3	CCR1,4,5	endothelial cells	+	+	+	[42,47]
CCL4	CCR5	macrophages	+	+	-	[42]
CCL5	CCR1,3,5	MSCs, tumor cells, TAFs	+	+		[42,48]
CCL7	CCR1,2,3		+	+		[42]
CCL8	CCR2,3,5		+	+		[42]
CCL11	CCR3		+	+		[42]
CCL12	CCR2		+	+		[42]
CCL16	CCR1		-	-		[49]
CCL17	CCR4	tumor cells, macrophages	-	+	+	[42,50]
CCL18	unknown	TAM		+		[42,51]
CCL19	CCR7,11	tumor cells, DCs	—	+	+	[42,52]
CCL20	CCR6	tumor cells	-	+	+	[53-56]
CCL21	CCR7,11	lymph nodes, tumor cells, endothelial cells	-	+		[57-64]
CCL22	CCR4	tumor cells, macrophages	-	+	+	[42,50,65]
CCL23	CCR1		+	+		[42]
CCL24	CCR3		+	+		[42]
CCL25	CCR9,11		+	+		[42]
CCL26	CCR3		+	+		[42]
CCL27	CCR2,3,10		+	+		[66]
CXCL1	CXCR1,2	tumor cells, TAFs	+	-		[59,67,68]
CXCL2	CXCR2	tumor cells, TAFs	+	+		[42,67-70]
CXCL3(GRO-α,β,γ)	CXCR2	tumor cells, TAFs	+	-		[67,68,71]
CXCL5( ENA-78)	CXCR1,2		+	+		[42,71,72]
CXCL6	CXCR1,2		+	+		[42]
CXCL7(NAP2)	CXCR1,2		+	+		[42]
CXCL8	CXCR1,2	TAFs,endothelial cells, tumor cells, monocytes	+	+		[19,42,59,73-76]
CXCL9	CXCR3		-			[77,78]
CXCL10	CXCR3	tumor cells	-			[59,78]
CXCL11	CXCR3		-			[61,79-82]
CXCL12	CXCR4	tumor cells, astrocytes, fibroblasts, microglia cells	+	+		[61,83-86]
CXCL13	CXCR5	tumor cells, macrophages,TAFs	+	+		[42,87-89]
CXCL14	unknown		-		+	[90]
CX3CL1	CX3CR1		+	+		[91-95]
PF4			-			[96,97]
IP-10	CXCR3		-			[38,71,80,98]
MIG	CXCR3		-	-		[71,99]

Abbreviations: +, promote; —, inhibit.

### How MSCs are recruited to tumors?

In normal status, MSCs natively present the tropism of adhering to matrix components, so they prefer to home to bone, lung and cartilage when injected intravenously. Nevertheless, a growing number of studies have shown that MSCs home to injury sites induced by inflammation without organ specificity [100-105]. MSCs migration to tumors is due to the tumor microenvironment accompanied by soluble factors produced by inflammaroty and tumor cells and chemokine receptors on MSCs. Those soluble inflammation-associated factors includes growth factors, chemokines and cytokines [17], such as epidermal growth factor (EGF), vascular endothelial growth factor-A (VEGF-A), fibroblast growth factor (FGF), platelet-derived growth factor (PDGF), hematopoietic growth factor (HGF), transforming growth factor-β1 (TGF-β1), tumor necrosis facror-α (TNF-α) [106-108], stromal cell-derived factor-1α (SDF-1α), IL-8, IL-6, granulocyte colony-stimulating factor (G-CSF), granulocyte-macrophage colony-stimulating factor (GM-CSF) [101], monocyte chemoattractant protein-1(MCP-1), urokinase-type plasminogen activator (uPA) [109].

Chemokine receptors expressed on MSCs such as CCR1, CCR4, CCR7, CCR9, CCR10, CXCR4, CXCR5, CXCR6, CX3CR1, and c-met lead to their tumor-homing process, too. Recent data implicate the hypoxia status, maintaining the chronic inflammation in tumor, also contribute to MSCs mobilization [110].

Based on the tumor-tropism property of MSCs, they can be used for tumor therapy as delivery vehicles of specific therapeutic genes. Transfering of IFN-β, IFN-γ, IL-2, IL-3, IL-12, CCL5, suicide gene cytocine deaminase (CD), adenovirus type 5 early-region 1A (Ad5.E1A) gene, tumor necrosis factor-related apoptosis-inducing ligand (TRAIL) into MSCs have been demonstrated to be anti-cancer, and transferring of other genes like CX3CL1 and NK4 also can inhibit metastases of tumors [11,111-124]. In addition, gene-enhanced MSCs are more effective for tissue repair and genetic disease treatment than unmodified MSCs [125-133].

### MSCs can promote tumor growth

A lot of functional studies have tackled the question of whether MSCs would influence tumor progression with respect of their tissue reparative function and immunosuppressive properties, however, it is still controversial. In osteosarcoma mouse model, MSCs promoting tumor growth was proved in both *in vivo* and *in vitro* study [134]. Similar results were also obtained from cancer cells co-implanted with MSCs in either colon carcinoma [135] or ovarian carcinoma [136]. However, the contrary effect was observed in Kaposi sarcoma (KS) model [135]. The reason for this is the difference in employing different doses of MSCs [137,138]. Nevertheless, MSCs are associated with tumor progression via shifting the balance of tissue microenvironment where they resided. Here, we will discuss their potential mechanisms in regulating tumor development.

#### MSCs promote angiogenesis in tumor

Blood Vessels are very important in tumor growth, especially at late stage of tumor progression. Current data suggested that MSCs promoting tumor angiogenesis was mainly dependent on their differentiation potential into endothelial-like cells or pericytes and secreting pro-angiogenic factors like vascular endothelial growth factor (VEGF), platelet-derived growth factor (PDGF), fibroblast growth factor (FGF) and CXCL12, thereby facilitating angiogenesis [1]. In addition, TAF, a critical component of tumor microenvironment, partly can be derived from MSCs that may be mobilized from local sites or circulation. In immunodeficiency mice, TAFs obtained from human tumor facilitate the growth of human breast and ovarian cancers via inhibiting tumor cell apoptosis, enhancing cell proliferation, as well as promoting angiogenesis [136].

#### MSCs suppress immune responses

Extensive investigations have shown that MSCs can exert immunosuppressive function to multiple types of immune cells from either innate immunity or adaptive immunity, such as T cells, B cells, DCs, NK cells and etc. [139]. For T cells, MSCs implemented inhibitory function through secreting high levels of chemokines and inhibitory factor, followed by decreasing T cell activity locally [91,140]. Moreover, MSCs were reported to suppress B cell function via inhibiting chemokine receptors expression [141], to prevent the maturation and cytokine production of DCs and to decrease IL-2 induced proliferation, cytokine production and cytotoxic activity of NK cells. Furthermore, MSCs can promote generation of T regulatory (Treg) cells [1,142]. The factors, such as prostaglandin E2 (PGE2), nitric oxide (NO), indoleamine 2,3-dioxigenase (IDO), PD-L1 and soluble HLA-G5, more or less, are involved in mediating MSC-based suppressive function directly or indirectly [1]. However, it is noteworthy that the immunosuppressive function of MSCs was, not innate, elicited by the synergy effect of interferon-γ (IFNγ) and any of three other proinflammatory cytokines, TNFα, IL-1α, or IL-1β [140].

#### MSCs inhibit apoptosis of tumor cells

Recent report has shown that serum-deprived MSCs could facilitate tumor growth and survival by autophagy [143] in both breast cancer animal model and *in vitro* assay. Tumor progression is accompanied with hypoxia and starvation, because solid tumors with size beyond 2 mm will limit tumor cells to uptake sufficient nutrient and oxygen due to less vasculature. Under hypoxia and starvation status, MSCs maintain their self-survival via autophagy, meanwhile, they release a lot of anti-apoptotic or pro-survival factors, such as VEGF, bFGF, PDGF, SDF-1α, insulin-like growth factor 1, 2 (IGF-1,2), transforming growth factor-β (TGF-β) and insulin-like factor binding protein-2 (IGFBP-2) [144-146] to prevent tumor cells from apoptosis and support their proliferation, while normal MSCs do not take this properties. VEGF can increase the Bcl-2/Bax ratio [147,148], bFGF can upregulate Bcl-2 expression [149], PDGF and TGF-β can induce the expression of VEGF and bFGF [150]. SDF-1α was repored to protect chronic lymphocytic leukemia (CLL) cells from apoptosis induced by drug [151]. Nitric oxide (NO), as another important molecule secreted by MSCs, was considered as a bifunctional regulator of apoptosis, proapoptotic at high dose and antiapoptotic at low [152]. Another essential chemokine IL-6 produced by tumor cells and MSCs inhibit apoptosis by upregulating the expression of Bcl-xl [153].

Another perspective also indicated that MSCs are the guardians of tumors, since they can mediate the chemotherapy resistance of tumor cells. Drug resistance was classified into environment mediated-drug resistance (EM-DR), cell adhesion mediated-drug resistance (CAM-DR) and soluble factor mediated-drug resistance (SM-DR), the latter two are associated with MSCs [154].

### MSCs can promote tumor metastasis

Metastasis is the major cause of cancer patient death. With more and more potential mechanisms of tumor metastasis are discovered, evidences from *in vitro* and *in vivo* studies both pointed out that MSCs have a close relationship with cancer metastasis [155-157]. MSCs induced metastasis only occurs in close proximity to tumor sites while the effect will be reversed when MSCs are inoculated in separate sites, even in nearby sites [156]. Other mechanisms, including epithelial-mesenchymal transition (EMT) induction, regulation of cancer stem cells (CSCs) and mesenchymal niches shifting, are also involved.

#### MSCs induce EMT of tumor cells

EMT was first identified as the characteristics of embryogenesis which was described as loss of cell adhesion, repression of E-cadherin expression, and increased cell mobility. The concept of EMT then was extended to tumor metastasis. In breast cancer, when tumor cells were co-cultured with MSCs or MSCs-conditioned medium, tumor cells and MSCs both can be induced to expressed EMT associated molecules [158,159]. Additional researches indicated that EMT appeared to be partly dependent on TGFβ and VEGF which are associated with MSCs [160,161].

#### MSCs regulate CSCs proliferation

Due to CSCs less sensitive to chemotherapy and toxins, they indeed play crucial roles in tumor metastasis [162]. MSCs can enhance CSCs proliferation by secreting cytokines, IL-6 and CXCL7, thereby facilitating the tumor growth [163-165].

#### MSCs shift mesenchymal niche

Another mechanism may be pointed to the mesenchymal niche. Growing evidences showed that MSCs can migrate not only to primary tumor sites but also to pre-metastatic sites [166-168]. Factors produced by primary tumors may diffuse to other tissues [167,168] and attract MSCs to be there, which will set up the mesenchymal niche for tumor cell migration. Further researches gave the indication that CCL5 produced by tumor cell-stimulated MSCs, through binding with CCR5, lead the tumor metastasis [156].

## Conclusion

This review draws attention to the complex of MSCs interaction with tumor microenvironment and highlights the fact that both tumor growth and tumor metastasis can be influenced by MSCs directly or indirectly. The effects of MSCs in tumor are varied: the notion that MSCs promoting tumor growth and metastasis has been supported from distinct aspects involved in angiogenesis, tumor cell survival, immunosuppressive microenvironment shape, as well as CSC maintenance and mesenchymal niche construction. However, the controversial results also exist. That can be attributing to the different microenvironment where they reside, the dose employed and their heterogeneity. Therefore, we should pay more attention to MSC-based therapy, especially the potential risk when it works as gene carriers. Nevertheless, it is important to understand the principles and mechanisms of MSCs regulating tumor progression that will give the indication how to employ MSCs to treat tumor.

## Abbreviations

MSCs: Mesenchymal stem cells; TAFs: Tumor-associated fibroblasts; TAM: Tumor-associated macrophage; CSCs: Cancer stem cells; PMN: Polymorphonuclear leukocytes; PGE2: Prostaglandin E2; IDO: Indoleamine 2,3-dioxigenase; NO: Nitric oxide; TGF-β: Transforming growth factor-β; TNF-α: Tumor necrosis factor-α; IFN-γ: Interferon-γ; IL-8: Interleukin-8; IGF-1,2: Insulin-like growth factor-1,2; IGFBP-2: Insulin-like growth factor binding protein-2; VEGF: Vascular endothelial growth factor; FGF: Fibroblast growth factor; PDGF: Platelet-derived growth factor; SDF-1α: Stroma-derived factor-1α; CD: Cytocine deaminase; Ad5.E1A gene: Adenovirus type 5 early-region 1A gene; TRAIL: Tumor necrosis factor-related apoptosis-inducing ligand; EGF: Epidermal growth factor; G-CSF: Granulocyte colony-stimulating factor; GM-CSF: Granulocyte-macrophage colony-stimulating factor.

## Competing interests

The authors declare that they have no competing interests.

## Authors’ contributions

X Y, J H, ZP H, Y W, C H, LX W and YF S planned the manuscript outline. X Y wrote the draft manuscript, J H, ZP H, Y W and C H revised the manuscript, LX W and YF S finalized the manuscript. All authors read and approve the final manuscript.
